# Pseudo-Synchronous Colorectal Cancer: A Case Report

**DOI:** 10.7759/cureus.73694

**Published:** 2024-11-14

**Authors:** Jose L Mejia, Luis A Mejia Sierra

**Affiliations:** 1 General Surgery, Ephrata Community Hospital/WellSpan Health, Ephrata, USA; 2 General and Vascular Surgery, Northwell Health, Long Island, USA

**Keywords:** colon cancer, metastatic colon cancer, mucinous colon cancer, right colon cancer, synchronous colon cancer

## Abstract

Colorectal cancer is one of the most common malignancies in men and women. Its metastatic pattern is predicted depending on the location, with aggressivity and local spread more common in the mucinous types. The incidence has been higher over the last decade due to increased screening. The most common type seen in clinical practice is sporadic, a somatic genetic disease that may be influenced by the local colonic environment and the individual's background genetic makeup. Synchronous cases are detected at the time of diagnosis or during the subsequent six months after an operation. There are no cases reported in the current literature of metastatic cecal cancer to the rectum, so the term "pseudo-synchronous" has been adopted to describe this process. The present case report involves an individual who presented with colon cancer and obstructive symptoms. In addition to the primary lesion at the cecum, another one was found at the rectum below a normal mucosa and, by definition, considered metastasic.

## Introduction

Colorectal cancer is the third most commonly diagnosed cancer in men and women and the third leading cause of death in the United States. Annually, there are approximately 104,000 colon cancers diagnosed and 45,000 rectal cancers [1]. More than 80% of new Colorectal cancers occur in patients over the age of 50, and according to the American Cancer Society, the lifetime risk of developing this disease is 1 in 23 for men and 1 in 26 for women. In the United States, the incidence and mortality are higher among African Americans, and risk factors include increased consumption of red and processed meat and animal fat. Inactivity, obesity, and smoking are associated with this cancer.

Around 60-65% of colon cancers are sporadic and caused by the accumulation of genetic changes brought along by various environmental factors; 5% of cases are related to inherited syndromes like familial adenomatous polyposis (<1%), hereditary nonpolyposis colorectal cancer or Lynch syndrome (2%-4%), and the remaining due to lower-level penetration variations in genes (<1%) and unknown hereditary genomic alterations [1]. The present report involves a metastasic lesion to the rectum from a primary cecal lesion.

## Case presentation

**Figure 1:**
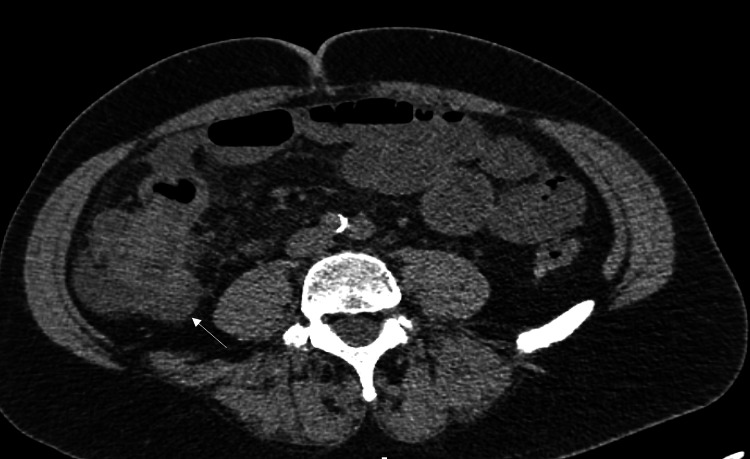
CT abdomen, axial cut, cecal mass (arrow)

We present a case report regarding a 59-year-old White male patient who, in 2018, was diagnosed with diffuse large B-cell lymphoma and treated with chemotherapy. He was followed by the oncology service and, in 2023, had a PET scan showing an avid lesion in the cecum. He was referred to General Surgery and was scheduled for a colonoscopy. During the procedure, he was found to have a large mass in the cecum, fungating and ulcerated, taking approximately sixty to seventy percent of the lumen; the colonoscope was slowly retracted, and no additional lesions were found in the rest of the colon. When inspecting the anal canal, a thick mucosal fold was identified at about 8-10 cm from the anal verge; macroscopically, the mucosa did not look distorted. Biopsies were taken from the two areas, and both came back positive for adenocarcinoma. A completion workup was done; a CT scan of the abdomen (Figure 1) disclosed a mass in the cecum with multiple liver lesions in both lobes and three sub-centimeter lesions in the lungs, two on the right and one on the left side. He had intermittent episodes of abdominal pain and constipation, requiring hospitalization in the last one. His lab work showed a hemoglobin of 14 mg/dl (n. 14-18 mg/dl), albumin of 3.3 g/dl (n. 3.4-5.4 g/dl), and CEA of 1,900 ng/ml (n. 0-2.9 ng/dl), with a normal Body Mass Index. 

After a long conversation with the patient and under the understanding that surgery would be palliative for him, a decision was taken to proceed. The rectal lesion was tattooed during the diagnostic colonoscopy. A mid-line laparotomy was performed, and the cavity was explored. There were multiple lesions in both lobes of the liver, and few implants were noted in the parietal peritoneum, including a small one close to the peritoneal reflection. The mass in the cecum was evident, and macroscopically, it was infiltrating the serosa of the colon. 

**Figure 2:**
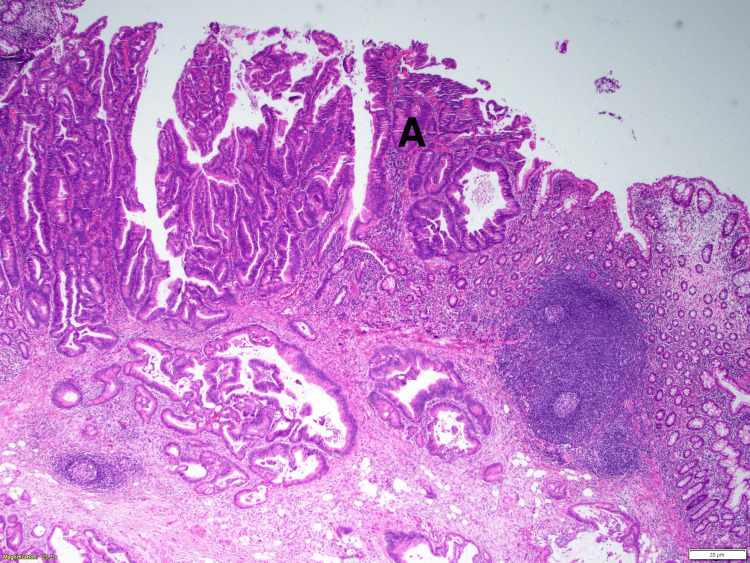
Cecal lesion with infiltration of the mucosa by tumor cells (A)

Biopsies were taken from all the relevant areas in the peritoneal cavity and liver, and a right hemicolectomy was performed with a side-to-side anastomosis. Then, after resecting the small implant by the peritoneal reflection and incising it to gain access to the rest of the rectum, a low anterior resection was performed; the rectal lesion was not in direct contact with the peritoneal implant, but about 6-8 centimeters from this area. We were able to achieve two centimeters of clean distal margin. A colorectal anastomosis was performed using an EEA (end-to-end anastomosis) stapler, and finally, a protective loop ileostomy was done. Drains were placed, and the cavity was closed. He was transferred to the floor and remained in the hospital for about one week. The pathology report indicated a 9.5 x 4.5 x 2.5 centimeters adenocarcinoma in the cecum, G2 moderately differentiated, with invasion into the visceral peritoneum (Figure 2). Seven of the 12 lymph nodes were positive, including all the implants harvested during the procedure in the peritoneum and the liver lesions. There was lymphovascular and perineural invasion; seven out of twelve lymph nodes were positive, and the margins were negative.

**Figure 3:**
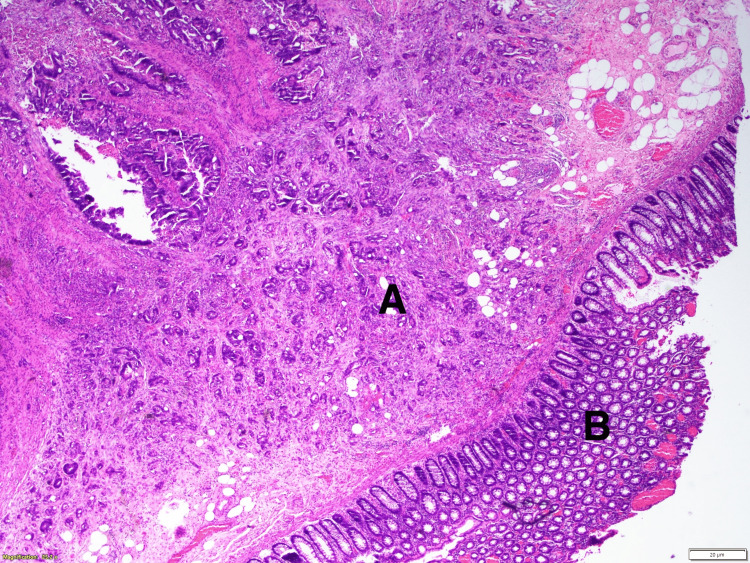
Rectal lesion, infiltration of tumor cells (A) under normal mucosa (B)

The lesion in the rectum, 1.5 x 1.5 x 1.4 centimeters, was located in the submucosa, without adenomatous changes and no distortion of the mucosal surface, and was pathologically considered to be metastatic (Figure 3).

The implant found by the peritoneal reflection during the surgery, by definition, was intrabdominal, small, and 6-8 centimeters away from the rectal lesion. Additional biomarkers were reported as MS stable, tumor mutational burden: 1 Muts/Mb, and there were genetic abnormalities in the KRAS, NRAS, APC, and TP53 genes. The final TNM was T4a, N2b, M1c, meaning a stage IVc.

In follow-up at the clinic, the patient had a satisfactory recovery; a port was placed two weeks later, and he was started on chemotherapy. At six months, he was doing well, and his ileostomy was reversed to improve his quality of life.

## Discussion

The “anatomical/mechanical” and “seed-and-soil” hypotheses are widely accepted to explain the metastatic spread. Research has also demonstrated that specific tumor cells may show a preference for particular target organs of metastasis [2].

Spread to the first drainage site may act as a “seed” to reach other organs further. From the colon and proximal rectum, blood is drained through the portal system to the liver, and from there, the next organ is the lungs via the heart. The distal rectum bypasses the liver, and the first organ encountered is the lungs. Additionally, we should remember that all organs in the gastrointestinal system share a common lymphatic drainage via the cisterna chyli to the left subclavian vein.

Regarding tumor biology, the invasion-metastasis cascade is the process that ends with the colonization of a distal organ and is described as a five-step ladder: (i) local invasion of tumor cells into the surrounding matrix, (ii) intravasation of these cells into the circulatory system, (iii) systemic transportation of tumor cells, (iv) extravasation of tumor cells in distal site, and (c) the colonization of distal organs and establishment of macroscopic tumors. The epithelial-mesenchymal transition (EMT) is a key complex program that enables stationary epithelial cells to lose cell-to-cell adherence and acquire mesenchymal properties essential for invasion and metastasis [3].

Synchronous colorectal cancer is a simultaneous occurrence of multiple primary tumors in the same patient within six months from the initial diagnosis. The overall prevalence is approximately 3.5%. The most common presentations are up to two or three primary lesions, and findings of four or more are highly rare [4].

Metastasis can also occur through the peritoneal fluid within the peritoneal cavity. It is more common with mucinous adenocarcinomas, which are generally more aggressive [5].

## Conclusions

It is not uncommon to find synchronous colorectal cancer, but metastasis of cecal cancer to the rectum is an infrequent occurrence. Despite systematic research, we have not been able to identify a similar case in the literature where the description of primary cancer in the colon has metastasized to itself. However, even when encountering this rare presentation, understanding the approach is a matter of semantics; in other words, two or more lesions must be treated with two or more resections following oncologic principles. It is still being determined at this time, with no previous cases of this kind reported, what will be the implication in the long term for the patient or if the adjuvant treatment needs to be modified to improve survival.
